# AdequacyModel: An R package for probability distributions and general purpose optimization

**DOI:** 10.1371/journal.pone.0221487

**Published:** 2019-08-26

**Authors:** Pedro Rafael D. Marinho, Rodrigo B. Silva, Marcelo Bourguignon, Gauss M. Cordeiro, Saralees Nadarajah

**Affiliations:** 1 Department of Statistics, Federal University of Paraíba, João Pessoa, Paraíba, Brazil; 2 Department of Statistics, Federal University of Rio Grande do Norte, Natal, Rio Grande do Norte, Brazil; 3 Department of Statistics, Federal University of Pernambuco, Recife, Pernambuco, Brazil; 4 School of Mathematics, University of Manchester, Manchester, United Kingdom; Griffith University, AUSTRALIA

## Abstract

Several lifetime distributions have played an important role to fit survival data. However, for some of these models, the computation of maximum likelihood estimators is quite difficult due to presence of flat regions in the search space, among other factors. Several well-known derivative-based optimization tools are unsuitable for obtaining such estimates. To circumvent this problem, we introduce the **AdequacyModel** computational library version 2.0.0 for the R statistical environment with two major contributions: a general optimization technique based on the Particle Swarm Optimization (PSO) method (with a minor modification of the original algorithm) and a set of statistical measures for assessment of the adequacy of the fitted model. This library is very useful for researchers in probability and statistics and has been cited in various papers in these areas. It serves as the basis for the **Newdistns** library (version 2.1) published in an impact journal in the area of computational statistics, see https://CRAN.R-project.org/package=Newdistns. It is also the basis of the **Wrapped** library (version 2.0), see https://CRAN.R-project.org/package=Wrapped. A third package making use of the **AdequacyModel** library can be found in https://CRAN.R-project.org/package=sglg. In addition, the proposed library has proved to be very useful for maximizing log-likelihood functions with complex search regions. The library provides a greater control of the optimization process by introducing a stop criterion based on a minimum number of iterations and the variance of a given proportion of optimal values. We emphasize that the new library can be used not only in statistics but in physics and mathematics as proved in several examples throughout the paper.

## 1 Introduction

In survival analysis, practitioners are usually interested in choosing the distribution providing the best fit from a broad class of candidate models. In this sense, lifetime distributions are continually evolving in parallel with computer-based tools, which allow for using more complex distributions with a larger number of parameters to better study sizable masses of data. The last two decades have been very prolific in generating new parametric models for lifetime data and several methods to generate new distributions can be found in the literature. In addition to extending traditional models, the relevance of new distributions relies on the fact that some of them can provide better fits to real data sets. For a survey on the most important recent lifetime distributions, the readers are referred to [1] and [2].

The main problem about recently proposed models is that in several cases one can obtain different solutions from different initial values when optimizing the corresponding likelihood functions, thus indicating the presence of flat regions in the search space. The term “flat” is used here to indicate that the minimum modulus of a function in a region is (in some sense) of the same order as the maximum modulus. In this case, most derivative-based optimization tools usually encounter difficulties such as getting trapped in local minima, which makes such approaches unsuitable to obtain the corresponding maximum likelihood estimates (MLEs). This is not, however, an exclusive problem of recent lifetime models. Several univariate and multivariate functions present the same issue. To circumvent this problem, some optimization algorithms based on swarm intelligence have been proposed over the last decades. This class of methods have shown simplicity, efficiency and robustness. One very popular swarm intelligence method is the Particle Swarm Optimization (PSO) for finding optimized solutions. The PSO is a stochastic search method introduced by [3] based on simple social behavior exhibited by birds and insects and, due to its simplicity in implementation, it has gained great popularity in optimization. It also has high level of convergence and low computational cost compared with other heuristic search methods. It traditionally uses a random sampling to find the optima, but it is superior, if compared with derivative-based methods, when the information about localization of the minimum (or maximum) is poor, which is the case when we have functions with flat regions. Further details about the PSO method can be found in [4].

Some variants of the PSO algorithm have been studied in the literature in order to fit different types of problems. [5] proposed a mirror-extended Curvelet transform and PSO to solve the problem of speckle noise and low contrast in Synthetic Aperture Radar images. Since data mining demands fast and precise partitioning of large data sets, they usually come with a wide range of attributes or features, requiring serious computational restrictions on the relevant clustering techniques. [6] presented an overview of PSO techniques for cluster analysis. The issue of choosing the most adequate values in the Support Vector Machine (SVM) methodology can be structured in terms of an optimization problem in order to minimize a prediction error. [7] introduced an integrated PSO algorithm (PSO + SVM) to solve this problem. [8] presented a PSO overview from a Bayesian perspective, providing a formal framework for incorporation of prior knowledge about the problem that is being solved. [9] adopted maximum likelihood via PSO algorithm to estimate the mixture of two Weibull distributions with complete and multiple censored data.

The main idea behind the proposed R package is to provide a set of tools for the assessment of the adequacy of lifetime models through a robust optimization method for determining the MLEs for lifetime distributions. Our contribution to the PSO algorithm is to replace the particles that eventually fall outside of the search region, which is a subtle variation of the original approach. By doing this, we expect to keep the viability of the algorithm and prevent all particles from converging to a local optimum. Furthermore, we provide more control over some aspects of the algorithm, such as the number of particles and iterations and a conditional stop criterion, based on a minimum number of iterations and the variance of a given proportion of optimal values. However, rather than focusing in the PSO itself, we provide an easy-to-use set of statistical measures to assess the adequacy of lifetime models for a given dataset. In addition to the MLEs, the package provides useful statistics to assess the goodness-of-fit of probabilistic models including Cramér-von Mises and Anderson-Darling statistics. These statistics are often used to compare non-nested models. The proposed package also gives other goodness-of-fit measures such as the Akaike information criterion and Bayesian information criterion, as well as the Kolmogorov-Smirnov test, through the goodness.fit() function. Even though our focus lies in lifetime models, the proposed optimization package can be used in several other areas as proved in some examples throughout the paper.

This paper is organized as follows. Section 2 describes some theoretical background of swarm intelligence and general ideas underlying the PSO approach. Section 3 presents the PSO algorithm designed for the **AdequacyModel** package. Section 4 provides practical examples showing effectiveness of our PSO algorithm compared to results from other techniques, especially those based on derivatives. Section 4 contains an application using real (not simulated) data. In Section 5, a Monte Carlo simulation study is presented to verify the behavior of the optimization obtained by the pso() function provided by the package. Finally, Section 6 gives concluding remarks on the main findings of the paper and the current package usage.

## 2 Conceptual design of the framework

### 2.1 Swarm intelligence

Swarm intelligence is an exciting research field still in its infancy compared to other paradigms in artificial intelligence. It is a branch of artificial intelligence concerned to the study of collective behavior of decentralized and self-organized systems in a social structure. These kinds of systems are composed by agents that interact in a small organization (swarm) wherein each individual is a particle. The main idea behind swarm intelligence is that an isolated particle has a very limited action in searching an ideal point for the solution of an nondeterministic polynomial (NP) time complete problem. However, the joint behavior of the particles in the search region shows evidence of artificial intelligence, i.e., the ability to take decisions to respond to changes. In this sense, the swarm intelligence concept arises directly from nature and is based on, for example, the self-organizing exploratory pattern of the schools of fish, flocks of birds and ant colonies. This collective behavior can not be described simply by aggregating the behavior of each element. Such situations have encouraged practitioners to obtain a satisfactory effect in the search for solutions to complex problems by studying methods that promote intelligent behavior through collaboration and competition among individuals. Swarm-based algorithms have been widely developed in the last decade and many successful applications in a variety of complex problems make them very promising, efficient and robust optimization tools, although very simple to implement. The idea is modeling very simple local interactions among individuals from which complex problem-solving behaviors arise.

### 2.2 Proposed PSO algorithm

The PSO algorithm is conceptually based on the social behavior of biological organisms that move in groups, such as birds and fishes. It has provided good solutions for problems of global optimization of box-constrained functions. The fundamental component of the PSO algorithm is a particle, which can move around in the search space in direction of an optimum by making use of its own information as well as that obtained from other particles within its neighborhood. The performance of a particle is affected by its fitness, which is evaluated by calculating the objective function of the problem to be solved. The particle’s movement in the search space is randomized. For each iteration of the PSO algorithm, the leader particle is set by minimizing the objective function in the corresponding iteration. The remaining particles arranged in the search region will randomly follow the leader particle and sweep the area around the leader particle. In this local search process, another particle may become the new leader and the other particles will follow the new leader randomly.

Mathematically, a particle *i* is featured by three vectors, namely:

- Its current location in the *n*-dimensional search space denoted by ***x***_*i*_ = (*x*_*i*1_, …, *x*_*in*_).
- The best individual position it has held so far denoted by ***p***_*i*_ = (*p*_*i*1_, …, *p*_*in*_).
- Its velocity ***v***_*i*_ = (*v*_*i*1_, …, *v*_*in*_).

Usually, the current location ***x***_*i*_ and velocity ***v***_*i*_ are initialized by sampling from uniform distributions throughout the search space and setting a maximum velocity value *v*_max_. Then, the particles move over the search space in sequential iterations driven by the following set of update equations:

- *v*_*i*,*d*_(*t* + 1) = *v*_*i*,*d*_(*t*) + *c*_1_ *r*_1_ [*p*_*i*,*d*_(*t*) − *x*_*i*,*d*_(*t*)] + *c*_2_ *r*_2_ [*p*_*g*,*d*_(*t*) − *x*_*i*,*d*_(*t*)];
- *x*_*i*,*d*_(*t* + 1) = *x*_*i*,*d*_(*t*) + *v*_*i*,*d*_(*t* + 1), *d* = 1, …, *n*,

where *c*_1_ and *c*_2_ are constants, *r*_1_ and *r*_2_ are independent uniform random numbers generated at every update along each individual direction *d* = 1, …, *n* and *p*_*g*_(*t*) is the *n*-dimensional vector of the best position encountered by any neighbor of the particle *i*. The velocities and positions at time *t* + 1 are influenced by the distances of the particle’s current location from its individual best historical experience *p*_*i*_(*t*) and its neighborhoods best historical experience *p*_*g*_(*t*) in a cooperative way.

The proposed PSO algorithm is a small modification of the standard PSO algorithm pioneered by [3], where f:R↦R with $R\subseteq R^{n}$ is the objective function to be minimized, *S* is the number of particles of the swarm (set of feasible points), each particle having a location vector $x_{i}\in R$ in the search space and a velocity vector defined by $v_{i}\in R$. Let *p*_*i*_ be the best known position of the particle *i* and *g* the best position of all particles. The small modifications are highlighted in the algorithm below. The default optimization does not address the optimization problem restricted to a region R. In the course of the iterations, it is common for several particles to fall outside the search region R. The strategy of eliminating these particles and randomly relocating them in the search region increases the viability of the algorithm by preventing all particles from converging to a local minimum.

1. For each particle *i* = 1, …, *S* do:

- Initialize the particle’s position with a uniformly distributed random vector: *x*_*i*_ ∼ *U*(*b*_*lo*_, *b*_*up*_), where *b*_*lo*_ and *b*_*up*_ are the lower and upper boundaries of the search-space.
- Initialize the particle’s best known position to its initial position: $p_{i}↢x_{i}$.
- If *f*(*p*_*i*_) < *f*(*g*) update the swarm’s best known position: $g↢p_{i}$.
- Initialize the particle’s velocity: *v*_*i*_ ∼ *U*(−|*b*_*up*_ − *b*_*lo*_|, |*b*_*up*_ − *b*_*lo*_|).

2. Until a termination criterion is met (e.g. number of iterations performed, or a solution with adequate objective function value found), repeat:

- For each particle *i* = 1, …, *S* do:
  - Pick random numbers: *r*_*p*_, *r*_*g*_ ∼ *U*(0, 1).
  - For each dimension *d* = 1, …, *n* do:
    - Update the particle’s velocity: $v_{i,d}↢\omega \;v_{i,d}+\varphi_{p}r_{p}\left(p_{i,d}-x_{i,d}\right)+\varphi_{g}r_{g}\left(g_{d}-x_{i,d}\right)$.
  - Update the particle’s position: $x_{i}↢x_{i}+v_{i}$.
  - If $x_{i}\notin R$
    - Eliminate *x*_*i*_.
    - Generate new values $x_{i}\in R$ (random values).
  - If *f*(*x*_*i*_) < *f*(*p*_*i*_) do:
    - Update the particle’s best known position: $p_{i}↢x_{i}$.
    - If *f*(*p*_*i*_) < *f*(*g*) update the swarm’s best known position: $g↢p_{i}$.

3. Now *g* holds the best found solution.

The parameter *ω* is called inertia coefficient and, as the name implies, controls the inertia of each particle arranged in the search space. The quantities φ_*p*_ and φ_*g*_ control the acceleration of each particle and are called acceleration coefficients. The PSO algorithm described above and implemented in the R programming language is given in the next section. A conditional stopping criterion will be discussed.

The choices of constants *ω*, φ_*p*_ and φ_*g*_ can dramatically affect the performance of the algorithm in the optimization process. Discussions about appropriate parameter choices have been the subject of some research, see [4] and [10].

One possible method is not to assess the fitness of the particles outside the search region and expect that these particles return to the search region due to some social interaction with other particles, see [11]. However, many problems involving likelihood-based inference require numerical constrained optimization. For example, the log-likelihood function is maximized subject to the constraint that the parameter of interest takes on the null-hypothesized value in the likelihood ratio test. In such problems, replacing the particles outside the feasible search region is a way to keep the viability of the algorithm.

## 3 The AdequacyModel package

### 3.1 Multi-parameter global optimization

The above algorithm has been implemented in the **AdequacyModel** package available in the R website. It is quite general and can be applied to maximize or minimize any objective function involving restriction vectors delimiting the search space. We want to make clear that the PSO function is constructed to minimize an objective function. Maximizing *f* is equivalent to minimizing −*f*. A brief description of the **AdequacyModel** package is as follows:

- func: an objective function to be minimized;
- S: number of considered particles. By default, the number of particles is equal to 150;
- lim_inf and lim_sup: define the inferior and superior boundaries of the search space, respectively;
- e: current error. The algorithm stops if the variance in the last iterations is less than or equal to e;
- data: by default data = NULL. However, when the func is a log-likelihood function, data is a data vector;
- N: minimum number of iterations (default N = 500);
- prop: Proportion of the last optimal values whose variance is computed and used as a stop criterion. If the number of iterations is greater than or equal to the minimum number of iterations N, then compute the variance of the last optimal values, where 0 ≤ prop ≤ 1.

One advantage of the PSO method is that we do not need to be concerned with initial values. Problems with initial values are frequent in iterative methods such as the BFGS when the objective function involves flat or nearly flat regions. We can obtain totally different results depending on the chosen initial values. This issue is not usual in heuristic-based methods, where the updated steps include randomness (generation of pseudo-random numbers). The following example presents issues related to initial guesses for the algorithm and shows the use of the pso function, especially when the argument func specifies the objective function. In order to provide a greater control of the optimization process, we define *N* as the stop criterion giving the minimum number of iterations. The number of optimal values considered for computing the variance is given by the proportion of optimal values given by the argument prop, which is equal to 0.2 by default. In other words, if 20% of the last optimal values has variance less than or equal to e, the algorithm will stop the global search, thus indicating convergence according to the fixed criteria. These stop criteria indicate that there is no significant improvements in the global search for this proportion of iterations. Thus, if the variance is less than or equal to *ε* > 0 assigned to the argument e of the pso() function, the algorithm will stop the iterations and return the best point that minimizes the objective function.

### 3.2 Examples

#### 3.2.1 Trigonometric function

**Fig 1:**
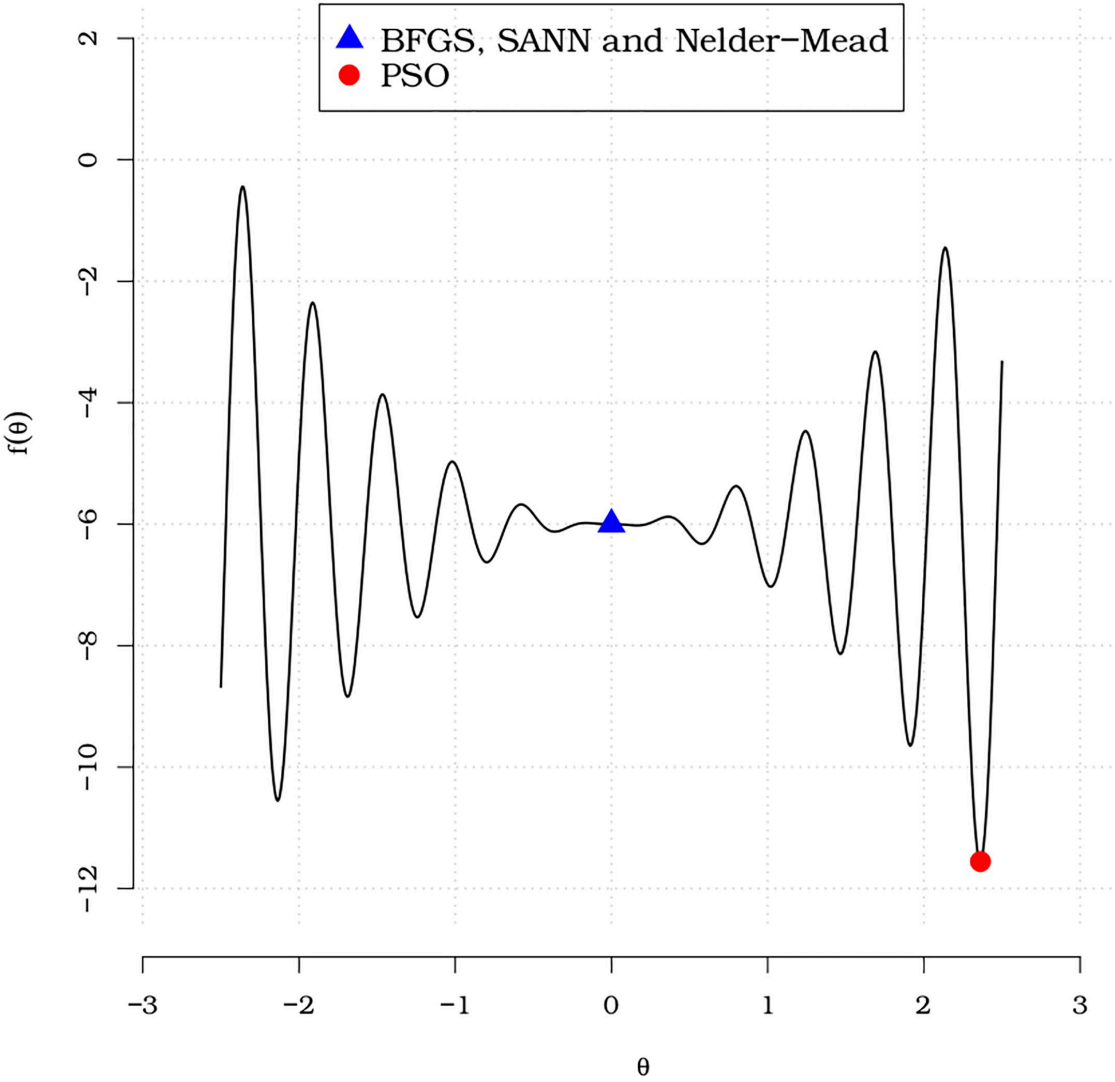
*f*(*θ*) = 6 + *θ*^2^ sin(14*θ*) with global minimum estimates.

Initially, we consider the case of a global search in a univariate function to estimate a one-dimensional vector. Consider the objective function *f*(*θ*) = 6 + *θ*^2^ sin(14*θ*). This function has local minima at *θ* = 2.3605 which globally minimizes *f*(*θ*) with *f*(2.3605) = −11.5618. Fig 1 plots *f*(*θ*) over *θ* ∈ [−2.5, 2.5]. The blue square symbol indicates the global minimum obtained by the BFGS, SANN and Nelder-Mead methods. The red bullet represents the global minimum obtained by the PSO method.

R> f <- function(x){

+  -(6 + x ^ 2 * sin(14 * x))

+ }

R> f_pso <- function(x, par){

+  theta <- par[1]

+  -(6 + theta ^ 2 * sin(14 * theta))

+ }

R> set.seed(9)

R> result_pso_f <- pso(func = f_pso, S = 500, lim_inf = -2.5,

+             lim_sup = 2.5, e = 0.0001)

R> set.seed(9)

R> result_sann_f <- optim(par = 0, fn = f, lower = -2.5, upper = 2.5,

+                method = “SANN”)

R> result_bfgs_f <- optim(par = 0, fn = f, lower = -2.5, upper = 2.5,

+                method = “BFGS”)

R> result_nelder_f <- optim(par = 0, fn = f, lower = -2.5, upper = 2.5,

+                method = “Nelder-Mead”)

Note that the global minimum estimates obtained by the BFGS, SANN and Nelder-Mead methods through the optim() function (for more details, execute ?optim) are heavily influenced by initial values. It is quite clear from Fig 1 that there is a *ε* > 0 such that *f* has derivative close to 0 around (−*ε*, *ε*). On the other hand, the pso function from the **AdequacyModel** script provides the true global minimum, which obviously coincides with the analytic solution. Note that all evaluated methods converge according to their associated stop criteria. For the BFGS, SANN and Nelder-Mead methods, we set the initial value as 0. For the SANN method and pso function, which involve randomization, we set a seed equal to 9, i.e. set.seed(9). The global minimum values obtained by the BFGS, Nelder-Mead and SANN methods are identical and influenced by the starting values. Unlike these methods, the PSO method implemented by the pso() function does not require initial values. These results can be replicated using the **AdequacyModel** package and the examples that follow. There is no need for initial information for optimizations through the pso() function.

#### 3.2.2 Easom function

**Fig 2:**
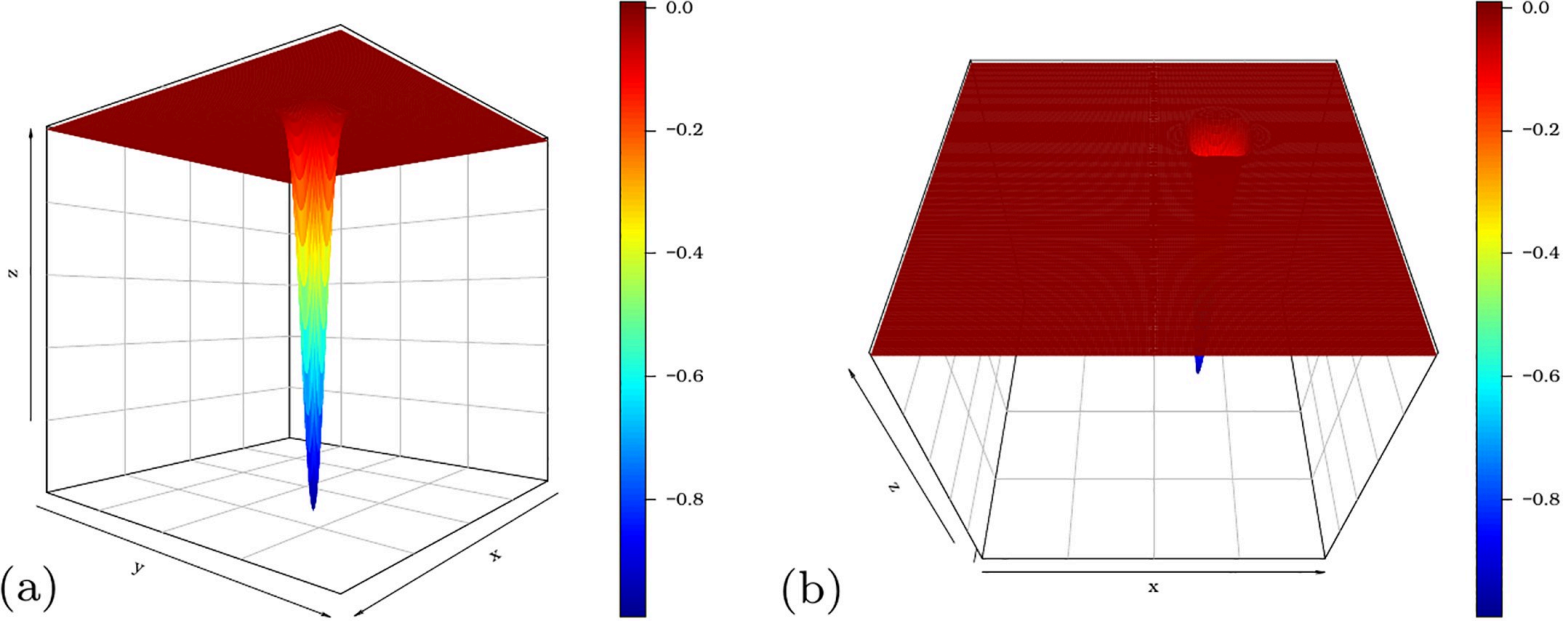
Easom function from two different angles.

We now consider the Easom function *f*(*x*, *y*) = −cos(*x*) cos(*y*) exp{−[(*x* − *π*)^2^ + (*y* − *π*)^2^]} for −10 ≤ *x*, *y* ≤ 10. Fig 2a and 2b display different angles of the function. The Easom function is minimized at *x* = *y* = *π* with *f*(*π*, *π*) = −1. The pso() function to minimize *f*(*x*, *y*) is

R> easom <- function(x, par){

+  x1 <- par[1]

+  x2 <- par[2]

+  -cos(x1) * cos(x2) * exp(-((x1 − pi) ^ 2 + (x2 − pi) ^ 2))

+ }

R> set.seed(9)

R> results_pso <- pso(func = easom, S = 500, lim_inf = c(-10, -10),

+            lim_sup = c(10, 10), e = 0.0001)

**Fig 3:**
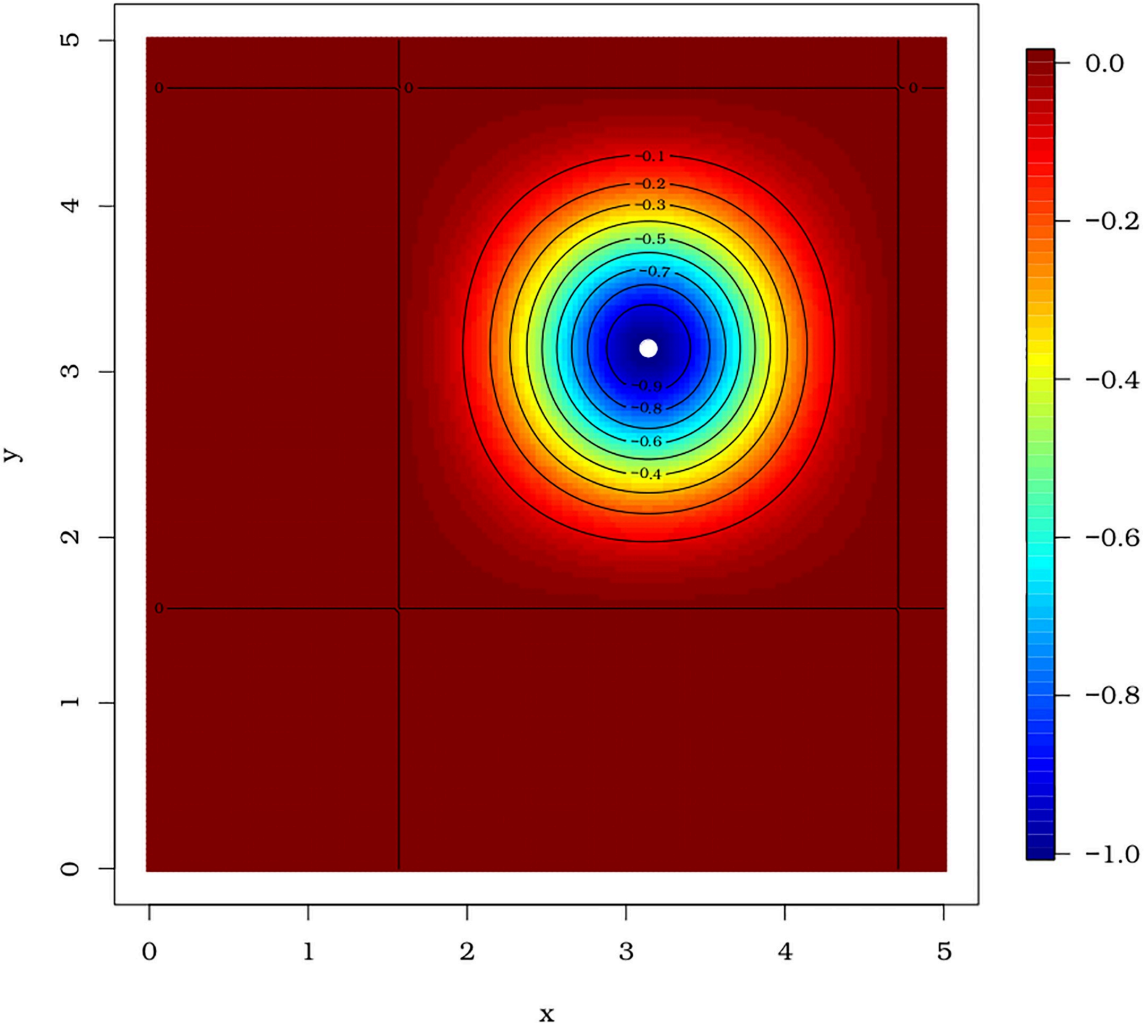
Levels of the Easom function. The white point is the minimum value obtained by the pso() function.

Before execution of the pso function, we set set.seed(9), for which the same results can be replicated. The estimated minimum points of the pso function are $\hat{x}=3.139752$ and $\hat{y}=3.141564$, which are very close to *x* = *y* = *π*. The convergence of the algorithm for values very close to the global optimum can be noted from the level curves in Fig 3.

We use the BFGS method through the optim() function and take initial values as *x* = −9 and *y* = 9. Note that the convergence is achieved in the BFGS method and the estimated minimum points coincide with the fixed initial values ($\hat{x}=-9$ and $\hat{y}=9$), which is quite different from the true minimum point *x* = *y* = *π*. Thus, the BFGS method is very sensitive to initial values. The reader can observe this fact from the code below.

R> easom1 <- function(x){

+  x1 <- x[1]

+  x2 <- x[2]

+  -cos(x1) * cos(x2) * exp(-((x1 − pi) ^ 2 + (x2 − pi) ^ 2))

+}

R> result_bfgs_easom <- optim(par = c(9, 9), fn = easom1, method = “BFGS”)

Notice that result_bfgs_easom$convergence == 0 equal to TRUE indicates convergence. Execute help(optim) for more details about the convergence criterion of the BFGS method implemented in the optim function. For the Easom function, convergence is harmed by the existence of infinite candidates for the minimum point distributed over a flat region. The output stored in the object result_bfgs_easom is presented below:

R> result_bfgs_easom

$par

[1] -9 9

$value

[1] -1.283436e-30

$counts

function gradient

1   1

$convergence

[1] 0

$message

NULL

Setting result_nelder_easom <- optim(par = c(-9, 9), fn = easom1, method = “Nelder-Mead”), we also obtain a distant point from the true global minimum point, where $\hat{x}=-8.1$ and $\hat{y}=9$ giving a minimum value approximately equal to zero. The results stored in result_nelder_easom are given below:

R> result_nelder_easom

$par

[1] -8.1 9.0

$value

[1] -3.609875e-71

$counts

function gradient

3   NA

$convergence

[1] 0

$message

NULL

A similar fact based on the simulated method can be found in the script below:

R> set.seed(9)

R> result_sann_easom <- optim(par = c(-9, 9), fn = easom1,

+          method = “SANN”)

As in the previous cases, we note that result_sann_easom$convergence == 0 is TRUE (there is convergence) and the estimated minimum point has coordinates distant from the coordinates of the true minimum point. The estimated coordinates are $\hat{x}=1.110688$ and $\hat{y}=13.934928$ with the seed fixed at 9, i.e. set.seed(9).

#### 3.2.3 Cross-in-tray function

**Fig 4:**
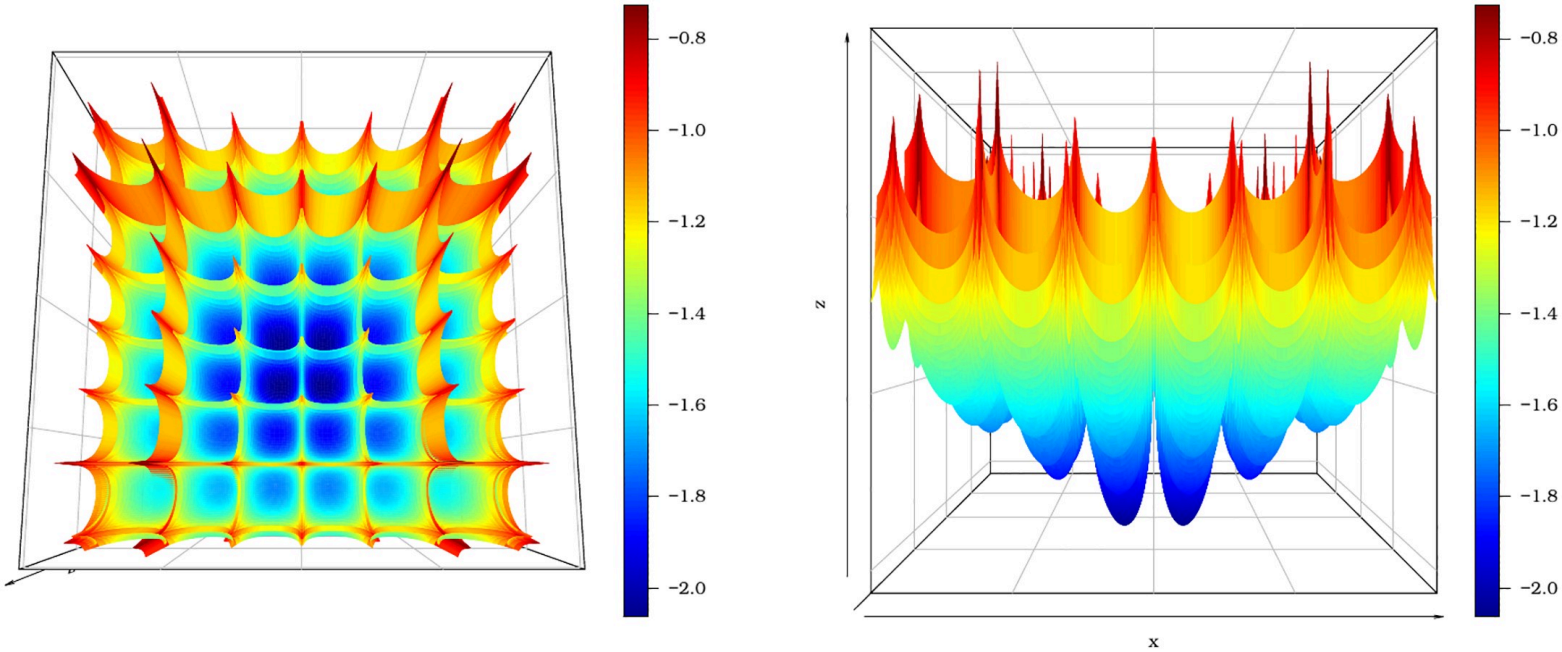
Cross-in-tray function from two different angles.

Now, we use the pso function to minimize the Cross-in-tray function. This is a difficult function to be minimized for different reasons from those presented in the previous examples. The Cross-in-tray function has many local minima as can be seen in Fig 4a and 4b. This fact can certainly harm the convergence of various algorithms that search for a global optimum. The Cross-in-tray function is

$$\begin{array}{c} f\left(x,y\right)=-0.0001(|\text{sin}\left(x\right)\;\text{sin}\left(y\right)\;\text{exp}(|100-\frac{\sqrt{x^{2}+y^{2}}}{\pi }|)|+1{)}^{0.1}, \end{array}$$

where −10 ≤ *x*, *y* ≤ 10 and

$$\begin{array}{c} \text{Min}=\{\begin{array}{ccc} f(1.34941,\;-1.34941) & = & -2.06261\\ f(1.34941,\;1.34941) & = & -2.06261\\ f(-1.34941,\;1.34941) & = & -2.06261\\ f(-1.34941,\;-1.34941) & = & -2.06261. \end{array} \end{array}$$

This function has four points of global minimum. Any estimate of the minimum point $\left(\hat{x},\;\hat{y}\right)$ with a minimum value close to −2.0626 can be regarded as a good solution.

**Fig 5:**
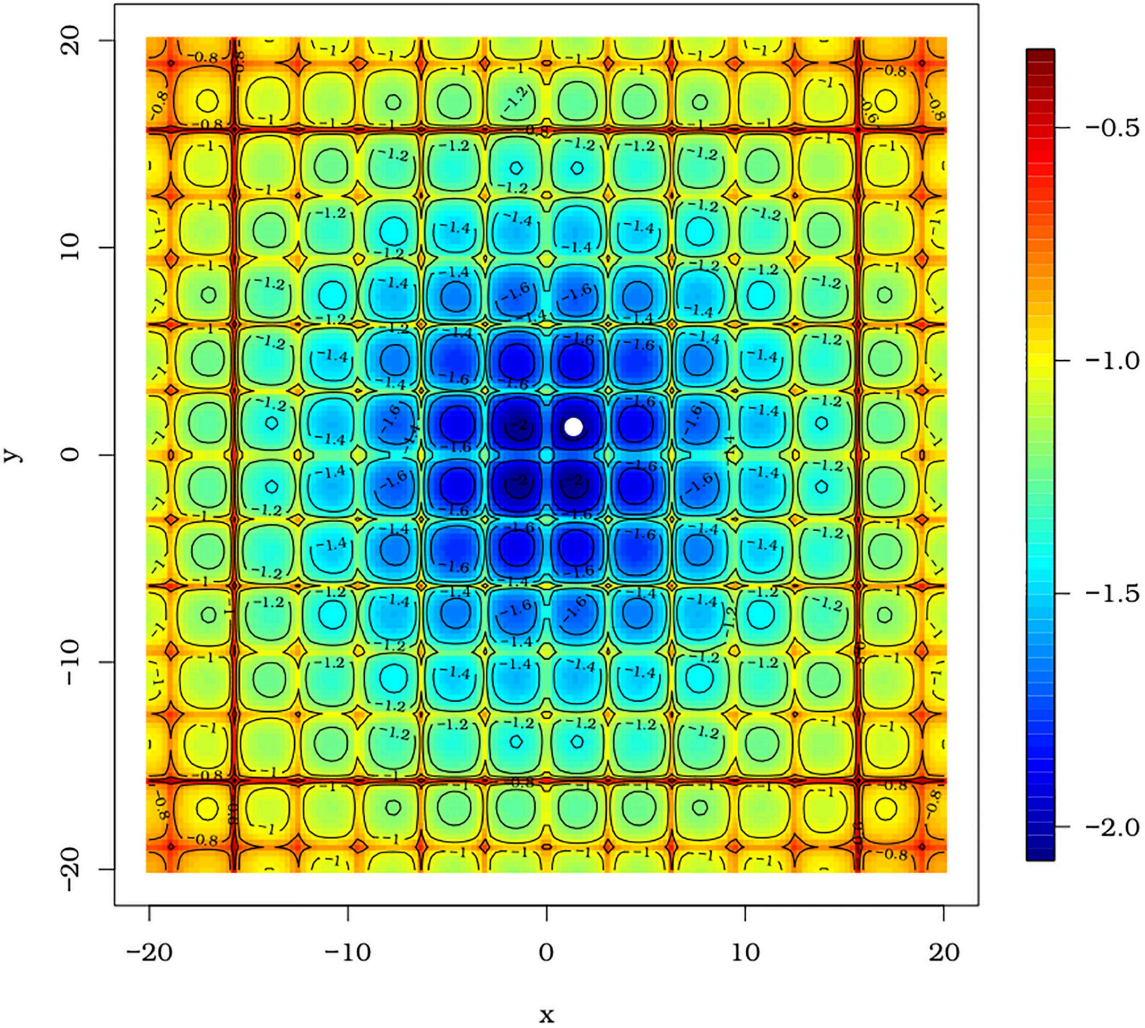
Levels of the Cross-in-tray function. The white point is the minimum value obtained by the pso() function.

**Note**: The results of the optimization using the optim() function and the Nelder-Mead, BFGS and simulated annealing methods can be determined from the code below. For all three methods, the initial value is chosen as (0, 0).

By means of the optim function, we note the convergence of the BFGS, SANN and Nelder-Mead methods with initial values at *x* = 0 and *y* = 0. The estimated values of *x* and *y* are equal to $\hat{x}=\hat{y}=0$ and $f(\hat{x},\hat{y})=-0.0001$ for the three approaches. The minimization of the Cross-in-tray function adopting the PSO algorithm achieves a satisfactory outcome as shown in Fig 5. The estimated minimum point is (1.3490, 1.3490) yielding the minimum value *f*(1.3490, 1.3490) = −2.0626. The results can be obtained with the script below:

R> cross <- function(x, par){

+  x1 <- par[1]

+  x2 <- par[2]

+  -0.0001 * (abs(sin(x1) * sin(x2) *

+         exp(abs(100 − sqrt(x1 ^ 2 + x2 ^ 2) / pi))) + 1) ^ 0.1

+ }

R> set.seed(9)

R> result_pso_cross <- pso(func = cross, S = 500, lim_inf = c(-10, -10),

+               lim_sup = c(10, 10), e = 0.0001)

R> cross1 <- function(x){

+  x1 <- x[1]

+  x2 <- x[2]

+  -0.0001 * (abs(sin(x1) * sin(x2) *

+        exp(abs(100 − sqrt(x1 ^ 2 + x2 ^ 2) / pi))) + 1) ^ 0.1

+ }

R> result_bfgs_cross <- optim(par = c(0, 0), fn = cross1, lower = -10,

+                upper = 10, method = “BFGS”)

R> result_nelder_cross <- optim(par = c(0, 0), fn = cross1, lower = -10,

+                upper = 10, method = “Nelder-Mead”)

R> set.seed(9)

R> result_sann_cross <- optim(par = c(0, 0), fn = cross1, lower = -10,

+                upper = 10, method = “SANN”)

#### 3.2.4 Hölder function

**Fig 6:**
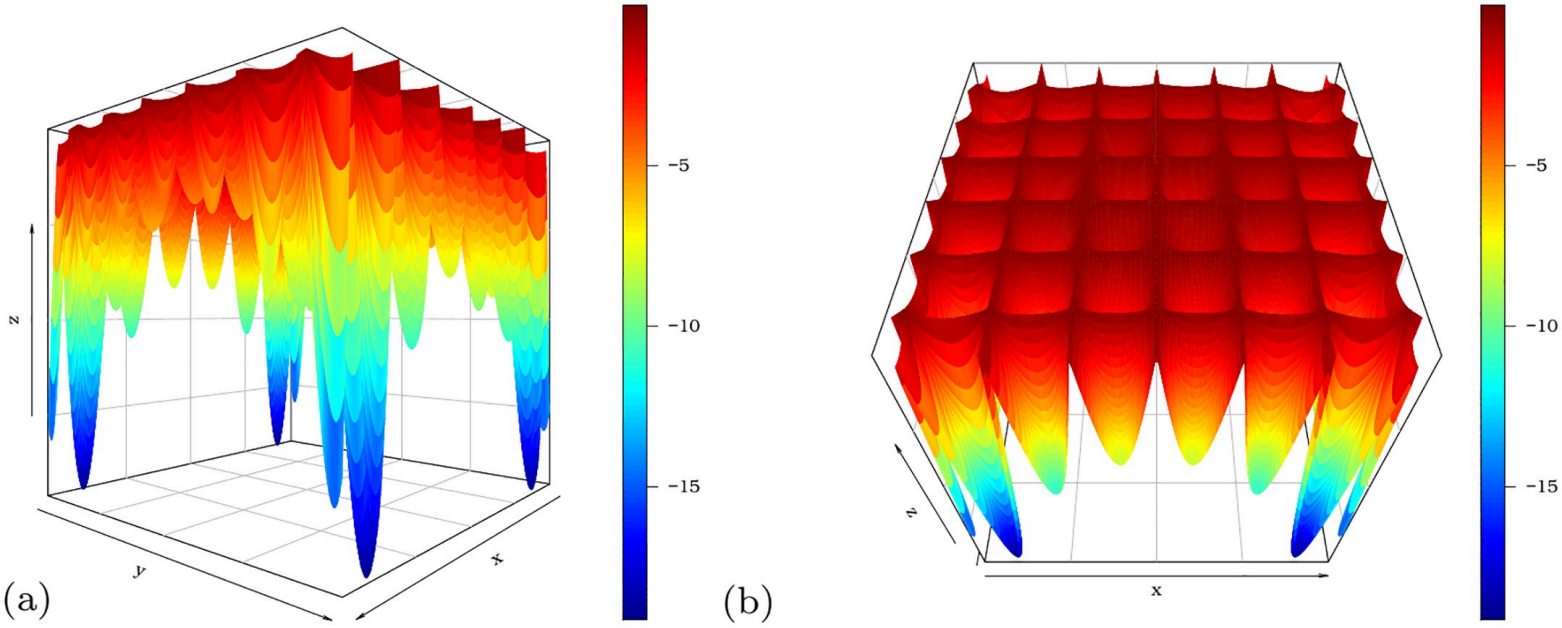
Hölder function at two different angles.

We consider the Hölder function, a very peculiar function that is difficult to be optimized, defined by

$$\begin{array}{c} f\left(x,y\right)=-|\text{sin}\left(x\right)\;\text{cos}\left(y\right)\;\text{exp}(|1-\frac{\sqrt{x^{2}+y^{2}}}{\pi }|)|, \end{array}$$

where

$$\begin{array}{c} \text{Min}=\{\begin{array}{ccc} f(8.05502,\;9.66459) & = & -19.2085\\ f(-8.05502,\;9.66459) & = & -19.2085\\ f(8.05502,\;-9.66459) & = & -19.2085\\ f(-8.05502,\;-9.66459) & = & -19.2085 \end{array} \end{array}$$

and −10 ≤ *x*, *y* ≤ 10. Fig 6 plots the Hölder function defined above.

**Fig 7:**
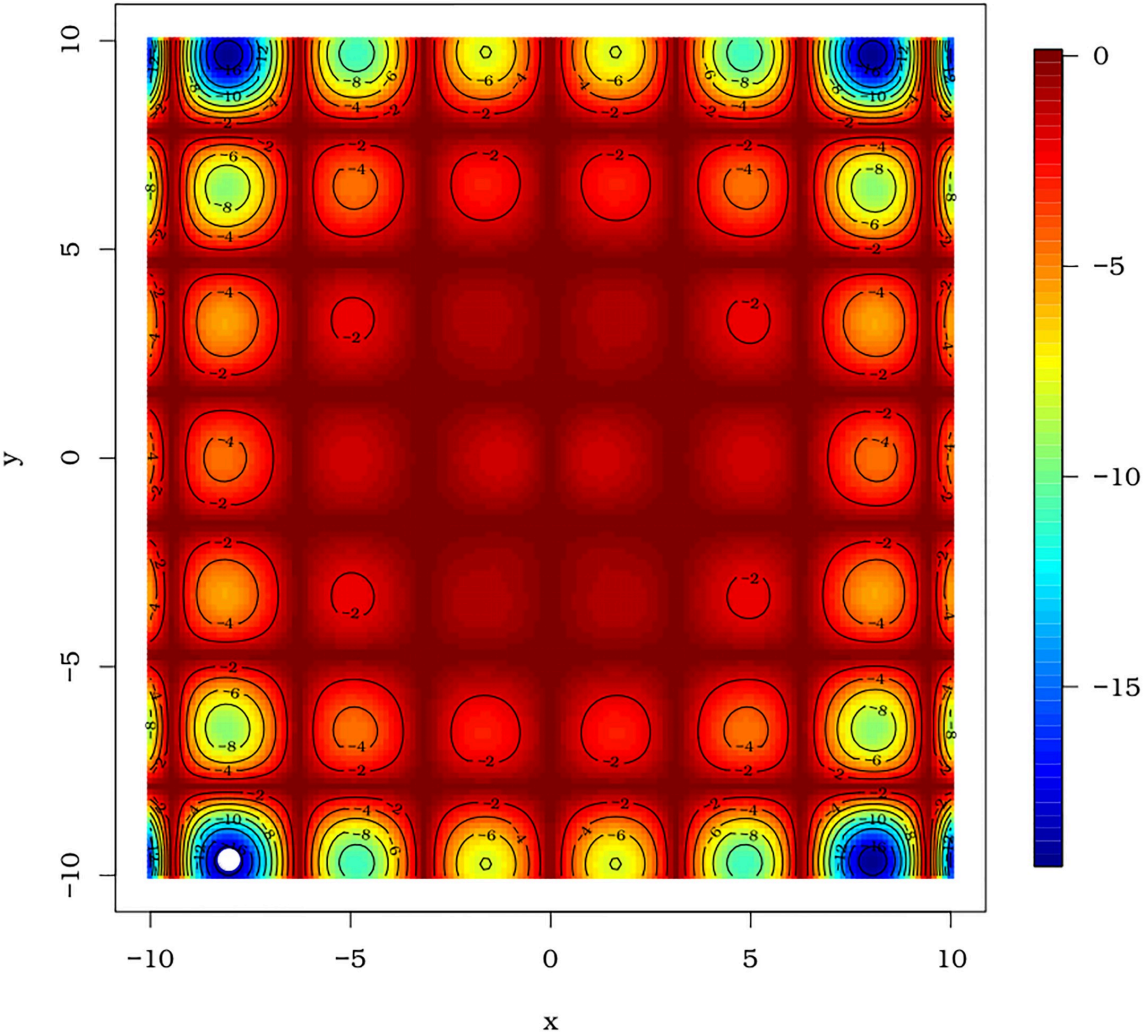
Levels of the Hölder function. The white point is the minimum value obtained by the pso() function.

For the Hölder function, the results obtained from the BFGS, SANN and Nelder-Mead methods, as in the previous examples, are not good. However, in all cases, there is convergence following the methods implemented in the optim() function. For initial values at (0, 0), the convergence leads to (0, 0), i.e., the three methods estimate the minimum point as $\hat{x}=0$ and $\hat{y}=0$. For the SANN method, we set set.seed(9). However, the problem is easily circumvented by increasing the number of iterations. Fig 7 plots the levels of the Hölder function versus the point of convergence of the PSO algorithm. We used the following script:

R> holder <- function(x, par){

+  x1 <- par[1]

+  x2 <- par[2]

+  -abs(sin(x1) * cos(x2) * exp(abs(1 − sqrt(x1 ^ 2 + x2 ^ 2) / pi)))

+ }

R> set.seed(9)

R> result_pso_holder <- pso(func = holder, S = 500,

+               lim_inf = c(-10, -10),

+               lim_sup = c(10, 10), e = 0.0001)

## 4 Fitting distributions with the AdequacyModel

The problem of deciding on the suitability of an unknown cumulative distribution function (cdf) *F*_*θ*_ from a sample *x*_1_, …, *x*_*n*_ is equivalent to the decision problem on an unknown parameter *θ*. Let $F=\{F_{\theta };\;\theta \in \Theta \}$ be a family of distributions, where Θ is the parameter space of *θ*. The best element *F*_*θ*_ in F can be determined from the MLE ${\hat{\theta }}_{n}$ of *θ*. Suppose there exists a *F*_*θ*_ for *F* evaluated at ${\hat{\theta }}_{n}$.

Some statistics are commonly used to verify the adequacy of the cdf *F*_*θ*_ to fit the observations. Alternatives to the likelihood ratio test were proposed by [12] by correcting the Cramér-von Mises (*W*^2^) and Anderson-Darling (*A*^2^) statistics. Let *F*_*n*_(*x*) be the empirical distribution function and $F(x;{\hat{\theta }}_{n})$ the postulated cdf evaluated at ${\hat{\theta }}_{n}$. According to [12], the usual Cramér-von Mises (*W*^2^) and Anderson-Darling (*A*^2^) statistics can be expressed as

$$\begin{array}{c} W^{2}=\sum_{i=1}^{n}{\left[u_{i}-\left\{\left(2i-1\right)/\left(2n\right)\right\}\right]}^{2}+1/\left(12n\right) \end{array}$$ (1)

and

$$\begin{array}{c} A^{2}=-n-n^{-1}\sum_{i=1}^{n}\left\{\left(2i-1\right)\;\text{log}\left(u_{i}\right)+\left(2n+1-2i\right)\;\text{log}\left(1-u_{i}\right)\right\}, \end{array}$$ (2)

respectively, where $u_{i}=\Phi \left(\left(y_{i}-\bar{y}\right)/s_{y}\right)$ (Φ is the standard normal cdf), $v_{i}=F\left(x_{i};{\hat{\theta }}_{n}\right)$, *y*_*i*_ = Φ^−1^(*v*_*i*_) and *s*_*y*_ is the sample standard deviation of the *y*_*i*_’s for *i* = 1, …, *n*.

The corrected statistics W* and A* are given by

$$\begin{array}{ccc} W^{*} & = & \{n\int_{-\infty }^{+\infty }{\left\{F_{n}\left(x\right)-F\left(x;{\hat{\theta }}_{n}\right)\right\}}^{2}dF\left(x;{\hat{\theta }}_{n}\right)\}(1+\frac{0.5}{n})=W^{2}(1+\frac{0.5}{n}), \end{array}$$ (3)

$$\begin{array}{ccc} A^{*} & = & \left\{n\int_{-\infty }^{+\infty }\frac{{\left\{F_{n}\left(x\right)-F\left(x;{\hat{\theta }}_{n}\right)\right\}}^{2}}{\left\{F\left(x;\hat{\theta }\right)\left(1-F\left(x;{\hat{\theta }}_{n}\right)\right)\right\}}dF\left(x;{\hat{\theta }}_{n}\right)\}(1+\frac{0.75}{n}+\frac{2.25}{n^{2}}\right)\\ & = & A^{2}(1+\frac{0.75}{n}+\frac{2.25}{n^{2}}). \end{array}$$ (4)

The statistics W* and A* measure the difference between *F*_*n*_(*x*) and $F(x;{\hat{\theta }}_{n})$. Low values of them provide evidence that $F(x;{\hat{\theta }}_{n})$ generates the data. The null hypothesis tested using (3) and (4) is that the random sample has the cdf *F*(*x*;*θ*). The algorithm below can be adopted to compute W* and A*:

1. Estimate *θ* by ${\hat{\theta }}_{n}$, order the observations to compute $v_{i}=F\left(x_{i};{\hat{\theta }}_{n}\right)$;
2. Compute *y*_*i*_ = Φ^−1^(*v*_*i*_), where Φ^−1^ is the standard normal quantile function;
3. Compute $u_{i}=\Phi \left\{\left(y_{i}-\bar{y}\right)/s_{y}\right\}$, where $\bar{y}=n^{-1}\sum_{i=1}^{n}y_{i}$ and $s_{y}^{2}={\left(n-1\right)}^{-1}\sum_{i=1}^{n}{\left(y_{i}-\bar{y}\right)}^{2}$;
4. Compute *W*^2^ and *A*^2^ using (1) and (2), respectively;
5. Compute W* = *W*^2^(1 + 0.5/*n*) and A* = *A*^2^(1 + 0.75/*n* + 2.25/*n*^2^), where *n* is the sample size;
6. Reject $H_{0}$ at the significance level *α* if the test statistics exceed the critical values presented by [12].

In practice, we can use W* and A* to compare two or more continuous distributions. The distribution that gives the lowest values of these statistics is the best suited to explain the random sample. The goodness.fit() function provides some useful statistics to assess the quality of fit of probabilistic models by including W* and A*. The function can also compute other measures such as the Akaike Information Criterion (AIC), Consistent Akaike Information Criterion (CAIC), Bayesian Information Criterion (BIC), Hannan-Quinn Information Criterion (HQIC) and Kolmogorov-Smirnov Test (KST). The general form for the function is given below:

goodness.fit(pdf, cdf, starts = NULL, data, method = “PSO”,

       domain = c(0, Inf), mle = NULL)

where

- pdf: probability density function (pdf);
- cdf: cumulative distribution function;
- starts: initial parameters to maximize the likelihood function;
- data: data vector;
- method: method used for minimization of the -log-likelihood function. The methods supported are: PSO (default), BFGS, Nelder-Mead, SANN, CG (conjugate gradient). We can also provide only the first letter of the methods, i.e., P, B, N, S or C, respectively;
- domain: domain of the pdf. By default the domain of the pdf is the open interval (0, ∞). This option must be a vector with two components;
- mle: vector with the MLEs. This option should be used if one already has knowledge of the MLEs. The default is NULL, i.e., the function will try to obtain the MLEs;
- …: If method = “PSO”, then all arguments of the pso() function could be passed to the goodness.fit() function.

It is not necessary to define the likelihood function or log-likelihood but only the pdf or the cdf. The function will check the validity of the arguments passed to goodness.fit(). For example, if the supplied functions to the arguments pdf or cdf are not genuine pdfs or cdfs, a message will be given so that the user can check the arguments passed. We provide below two examples of the use of the goodness.fit() function.

### 4.1 Carbon fiber data

Consider a data set of stress (until fracture) of carbon fibres (in Gba). The data can be obtained from [13]. The data and some details can be accessed with the command data(carbone) in the **AdequacyModel** package. Suppose also that we are interested in choosing the best model in $F=\{F_{\theta };\;\theta \in \Theta \}$ that can represent the distribution of observations in carbone. We suppose that F is exponentiated Weibull (Exp-Weibull) distributed with cdf

$$\begin{array}{c} F\left(x;\alpha ,\beta ,a\right)=\{1-\text{exp}[-{\left(\alpha x\right)}^{\beta }]{\}}^{a},\;\;x>0, \end{array}$$

where *α*, *β* and *a* are positive parameters. Thus, each element in F is of the form *F*(*x*; *α*, *β*, *a*). We initially implement the pdf *f*(*x*; *α*, *β*, *a*) and cdf *F*(*x*; *α*, *β*, *a*). They will serve as arguments for pdf and cdf, respectively. We present below the implementation of these functions input to the goodness.fit() function.

R> # Probability density function.

R> pdf_expweibull <- function(par, x) {

+  alpha <- par[1]

+  beta <- par[2]

+  a <- par[3]

+  alpha * beta * a * exp(-(alpha * x) ^ beta) * (alpha * x) ^ (beta

+  - 1) * (1 − exp(-(alpha * x) ^ beta)) ^ (a − 1)

+ }

R> # Cumulative distribution function.

R> cdf_expweibull <- function(par, x) {

+  alpha <- par[1]

+  beta <- par[2]

+  a <- par[3]

+  (1 − exp(-(alpha * x) ^ beta)) ^ a

+ }

R> data(carbone)

R> results <- goodness.fit(pdf = pdf_expweibull, cdf = cdf_expweibull,

+              starts = c(1, 1, 1), data = carbone,

+              method = “BFGS”, domain = c(0, Inf),

+              mle = NULL)

The object results features all goodness-of-fit statistics cited previously as well as the MLEs in case mle = NULL (default) and the standard errors of the MLEs if the argument method receives PSO, BFGS, Nelder-Mead, SANN or CG. Thus,

- R> results$W provides the statistic W*;
- R> results$A provides the statistic A*;
- R> results$KS provides the Kolmogorov-Smirnov statistic;
- R> results$mle provides a vector with the MLEs of the model parameters given as arguments for the pdf;
- R> results$AIC: provides the AIC statistic;
- R> results$CAIC: provides the CAIC statistic;
- R> results$BIC: provides the BIC statistic;
- R> results$HQIC: provides the HQIC statistic;
- R> result$KS: returns an object of class htest with information on the Kolmogorov-Smirnov test;
- R> results$Erro: provides the standard errors of the MLEs of the parameters, given as arguments for pdf and cdf;
- R> results$value: displays the minimum value of -log(likelihood);
- R> result$Convergence: provides information on the convergence of the method passed as an argument for method. If result$Convergence == 0 is TRUE, there was convergence.

In case method = “PSO” (default), the standard errors will not be provided. The researcher may obtain these standard errors using bootstrap, see [14]. We provide below the results stored in the object results (output of the goodness.fit() function) and a plot of the fitted Exp-Weibull pdf.

R> results

$W

[1] 0.07047089

$A

[1] 0.4133608

$KS

One-sample Kolmogorov-Smirnov test

data: data

D = 0.064568, p-value = 0.7987

alternative hypothesis: two-sided

$mle

[1] 0.3731249 2.4058010 1.3198053

$AIC

[1] 288.6641

$CAIC

[1] 288.9141

$BIC

[1] 296.4796

$HQIC

[1] 291.8272

$Erro

[1] 0.06265212 0.60467076 0.59835491

$Value

[1] 141.332

$Convergence

[1] 0

**Fig 8:**
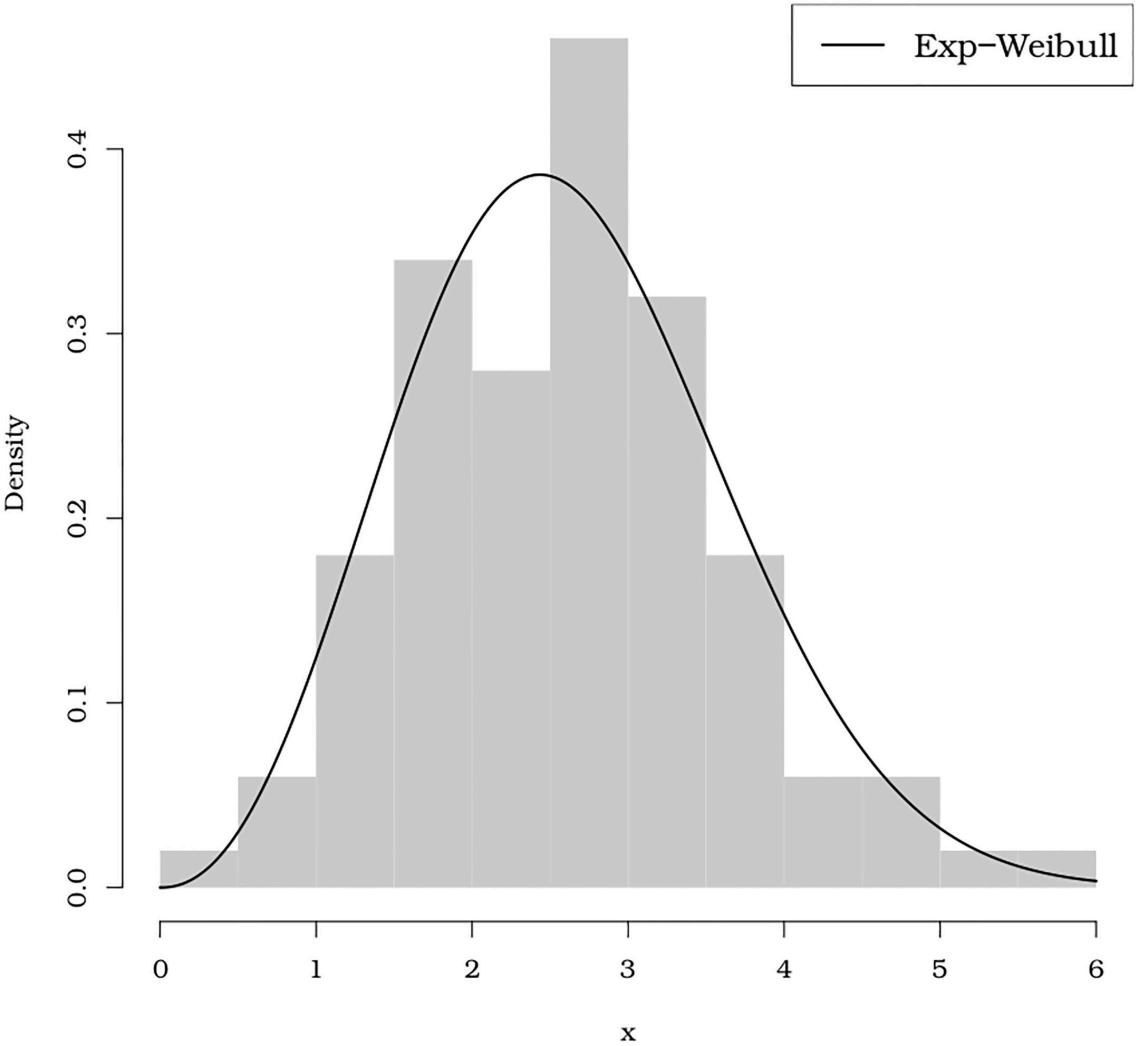
Fitted Exp-Weibull pdf to stress data (until fracture) of carbon fibers in Gba.

**Notes**: (*i*) The Kolmogorov-Smirnov statistic may return NA with a certain frequency, meaning that this statistic is not reliable for the current data. More details about this issue can be read from help(ks.test). In situations where results$Convergence==0 is TRUE, there is convergence for the method passed as an argument to method that minimizes the log-likelihood function multiplied by -1, that is, the method minimizes -log(likelihood). (*ii*) The convergence criterion as well as other details about possible values returned by results$Convergence can be obtained with help(optim) if the argument method of the goodness.fit() function receives the strings “BFGS”, “Nelder-Mead”, “SANN” or “CG” (or the initial letters “B”, “N”, “S” or “C”). For the PSO method (default method = “PSO”), the convergence criterion is displayed as discussed in Section 2, which is normally satisfied. (*iii*) The script for Fig 8 is:

R> pdf(file = “plot_adjustment.pdf”, width = 9, height = 9, paper = “special”,

+   family = “Bookman”, pointsize = 14)

x = seq(0, 6, length.out = 250)

hist(carbone, probability = TRUE, xlab = “x”, main = “”)

lines(x, pdf_expweibull(par = results$mle, x), lwd = 2)

legend(“topright”, legend = c(expression(paste(“Exp-Weibull”))), lwd = c(2.5),

+   inset = 0.03, lty = c(1), cex = 1.1, col = c(“black”))

dev.off()

### 4.2 Flood level data

As a second example, we shall analyse a data set from [15] which refers to 20 observations of the maximum ood level (in millions of cubic feet per second) for the Susquehanna River in Harrisburg, Pennsylvania. The data are: 0.26, 0.27, 0.30, 0.32, 0.32, 0.34, 0.38, 0.38, 0.39, 0.40, 0.41, 0.42, 0.42, 0.42, 0.45, 0.48, 0.49, 0.61, 0.65, 0.74. These data were fitted by using the Kumaraswamy beta (Kw-beta) distribution.

A random variable *X* follows a Kw-beta distribution with parameters *a*, *b*, *α*, *β* > 0 if its cdf and pdf are given by

$$\begin{array}{c} F\left(x;\alpha ,\beta ,a,b\right)=1-{\left\{1-G{\left(x;\alpha ,\beta \right)}^{a}\right\}}^{b} \end{array}$$

and

$$\begin{array}{c} f\left(x;\alpha ,\beta ,a,b\right)=a\;b\;g\left(x;\alpha ,\beta \right)G{\left(x;\alpha ,\beta \right)}^{a-1}{\left\{1-G{\left(x;\alpha ,\beta \right)}^{a}\right\}}^{b-1}, \end{array}$$

respectively, where *G*(*x*; *α*, *β*) = I_*x*_(*α*, *β*), *g*(*x*; *α*, *β*) = *x*^*α*−1^(1 − *x*)^*b*−1^/B(*a*, *b*), I_*x*_(*a*, *b*) denotes the incomplete beta function ratio ${\text{I}}_{y}\left(a,b\right)=\frac{1}{\text{B}(a,b)}\int_{0}^{y}\omega^{a-1}{\left(1-\omega \right)}^{b-1}\text{d}\omega$ and B(⋅, ⋅) denotes the beta function.

Obviously, due to the genesis of the Kw-beta distribution, the flood levels are ideally modelled by this distribution. Thus, the use of the Kw-beta distribution for fitting this data set is well justified. We present below the implementation of the functions input to the goodness.fit() function.

R> # Kumaraswamy Beta—Probability density function.

R> pdf_kwbeta <- function(par, x){

+  beta <- par[1]

+  a <- par[2]

+  alpha <- par[3]

+  b <- par[4]

+  (a * b * x ^ (alpha − 1) * (1 − x) ^ (beta- 1) *

+  (pbeta(x,alpha,beta)) ^ (a − 1) *

+  (1 − pbeta(x, alpha, beta) ^ a) ^ (b − 1)) / beta(alpha, beta)

+ }

R>

R> # Kumaraswamy Beta − Cumulative distribution function.

R> cdf_kwbeta <- function(par, x){

+  beta <- par[1]

+  a <- par[2]

+  alpha <- par[3]

+  b <- par[4]

+  1 − (1 − pbeta(x, alpha, beta) ^ a) ^ b

+ }

R>

R> # Data set

R> data_unit <- c(0.26, 0.27, 0.30, 0.32, 0.32, 0.34, 0.38, 0.38, 0.39,

+         0.40, 0.41, 0.42, 0.42, 0.42, 0.45, 0.48, 0.49, 0.61,

+         0.65, 0.74)

R>

R> results <- goodness.fit(pdf = pdf_kwbeta, cdf = cdf_kwbeta,

+               starts = c(1, 1, 1, 1), data = data_unit,

+               method = “BFGS”, domain = c(0,1),

+               lim_inf = c(0, 0, 0, 0),

+               lim_sup = c(10, 10, 10, 10), S = 200,

+               prop = 0.1, N = 40)

R> results

$‘W’

[1] 0.06228039

$A

[1] 0.3483813

$KS

   One-sample Kolmogorov-Smirnov test

data: data

D = 0.14992, p-value = 0.7596

alternative hypothesis: two-sided

$mle

[1] 28.3805432 29.0062276 5.2899143 0.1774844

$AIC

[1] -24.71882

$‘CAIC’

[1] -22.05215

$BIC

[1] -20.73589

$HQIC

[1] -23.94131

$Erro

[1] 1.93409776 30.74704316 1.92556208 0.04377468

$Value

[1] -16.35941

$Convergence

[1] 0

The estimates of the parameters are $\left(\hat{a},\hat{b},\hat{\alpha },\hat{\beta }\right)=\left(29.0062,0.1775,5.2899,28.3805\right)$, and the standard errors for the estimates $\hat{a},\hat{b},\hat{\alpha }$ and $\hat{\beta }$ are 30.747, 0.0438, 1.9256 and 1.9341, respectively.

### 4.3 TTT plot

Several aspects of an absolutely continuous distribution can be seen more clearly from the hazard rate function (hrf) than from either the cdf or the pdf. The hrf is an important quantity characterizing life phenomena. Let *X* be a random variable with pdf *f*(*x*) and cdf *F*(*x*). The hrf of *X* is defined by

$$\begin{array}{c} h\left(x\right)=\frac{f(x)}{1-F(x)}, \end{array}$$

where 1 − *F*(*x*) is the survival function.

The hrf may increase, decrease, be a constant, upside-down bathtub shaped, bathtub shaped or indicate a more complicated process. In many applications there is a qualitative information about the hazard rate shape, which can help in selecting a specified model. In this context, a device called the *total time on test* (TTT) or its scaled TTT transform proposed by [16] may be used for assessing the empirical behavior of the hrf. The scaled TTT transform is defined by (0 < *u* < 1)

$$\begin{array}{c} \phi_{X}\left(u\right)=\frac{H_{X}^{-1}\left(u\right)}{H_{X}^{-1}\left(1\right)}, \end{array}$$

where $H_{X}^{-1}\left(u\right)=\int_{0}^{Q(u)}\left[1-F\left(x\right)\right]dx$ and *Q*(*u*) is the quantile function of *X*. The quantity *ϕ*_*X*_(⋅) can be empirically approximated by

$$\begin{array}{c} T\left(i/n\right)=\frac{\sum_{k=1}^{i}X_{k:n}+\left(n-i\right)X_{i:n}}{\sum_{k=1}^{n}X_{k}}, \end{array}$$

where *i* = 1, …, *n* and *X*_*k*:*n*_, *k* = 1, …, *n*, are the order statistics of the sample. The TTT plot is obtained by plotting *T*(*i*/*n*) against *i*/*n*. The plot can detect the type of the hazard rate of the data. It is a straight diagonal for constant hazrd rates, it is convex for decreasing hazard rates and concave for increasing hazard rates. It is first convex and then concave if the hazard rate is bathtub-shaped. It is first concave and then convex if the hazard rate is upside-down bathtub. For more details, see [16].

The computation of the TTT plot is addressed in the **AdequacyModel** package. The real data set named carbone is used to illustrate the TTT plot function TTT() of this package. carbone refers to breaking stress of carbon fibres (in Gba) from [13]:

R> library(AdequacyModel)

R> data(carbone)

R> TTT(carbone, col = “red”, lwd = 2.5, grid = TRUE, lty = 2)

**Fig 9:**
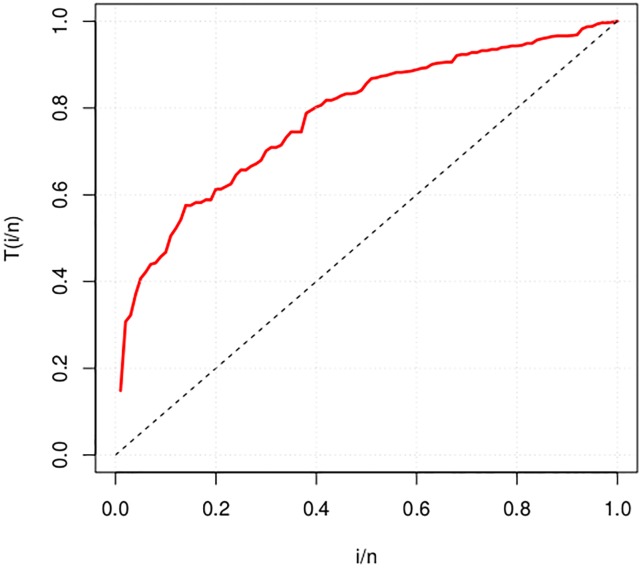
TTT-plot for carbon data.

The TTT plot for the carbone data [13] is displayed in Fig 9, which reveals an increasing hrf. This plot reveals that distributions with increasing hrf could be good candidates for modeling the carbone data, see the theoretical plot in Fig 1 in [16].

## 5 Simulations

In order to study the consistency of the PSO method, implemented in the pso() function of the **AdequacyModel** package, two Monte Carlo (MC) simulations are performed considering the Rastrigin and Himmelblau’s functions. All the results can be reproduced with the code in the Appendix A. The functions are quite peculiar, both being multimodal. Multimodal functions belong to a class of functions that impose great challenges on optimization methods.

The Rastringin function considered here is defined by −5.12 ≤ *x*_*i*_ ≤ 5.12, ∀*x*_*i*_ ∈ ***x***, such that

$$\begin{array}{c} f\left(x\right)=An+\sum_{i=1}^{n}\left[x_{i}^{2}-A\;\text{cos}\left(2\pi x_{i}\right)\right], \end{array}$$ (5)

where *A* = 10. Further, we take *n* = 2 in (5) in order to construct a three-dimensional chart. This function has the minimum value *f*(0, 0) = 0. On the other hand, the Himmelblau’s function is defined on −5 ≤ *x* ≤ 5, such that

$$\begin{array}{c} f\left(x,y\right)={\left(x^{2}+y-11\right)}^{2}+{\left(x+y^{2}-7\right)}^{2}. \end{array}$$ (6)

This function has four global minimum points, with zero being the global minimum value. The four global minimum points are:

$$\begin{array}{c} \text{Min}=\{\begin{array}{ccc} f(3.0,2.0) & = & 0.0\\ f(-2.805118,3.131312) & = & 0.0\\ f(-3.779310,-3.283186) & = & 0.0\\ f(3.584428,-1.848126) & = & 0.0 \end{array} \end{array}$$

**Fig 10:**
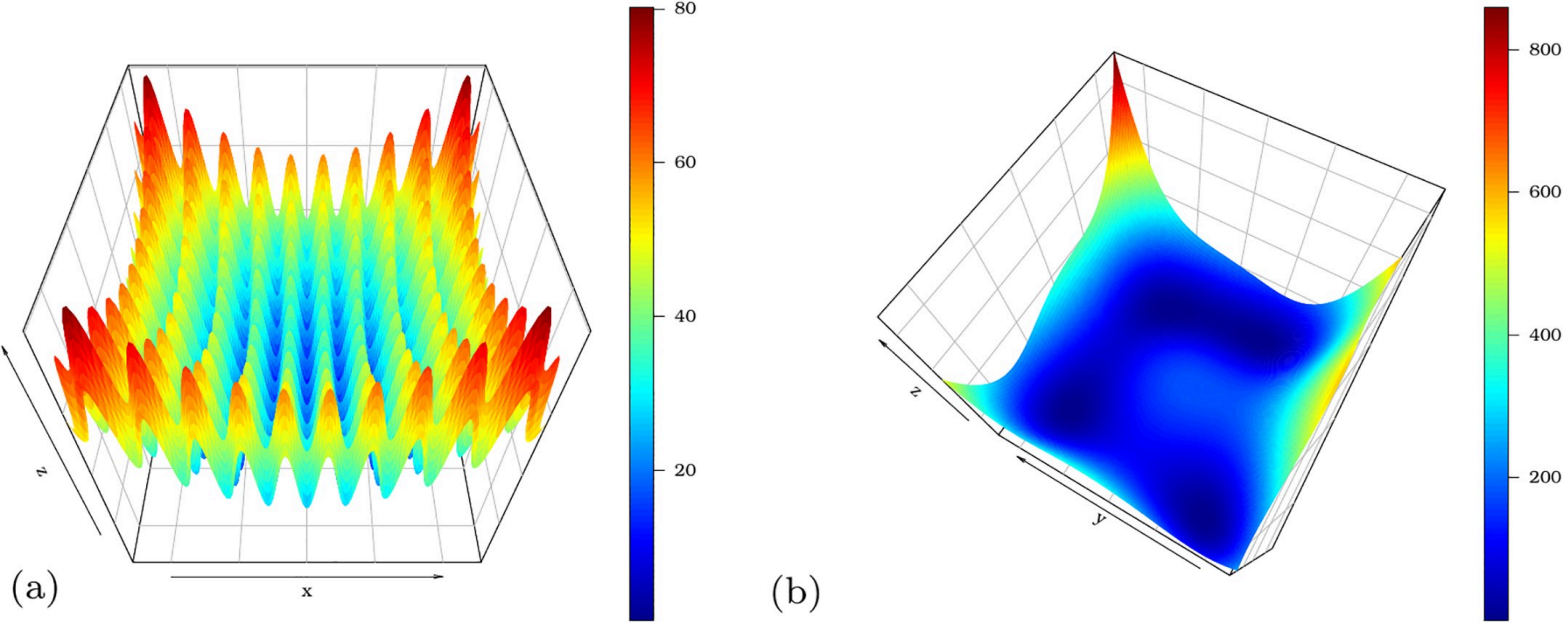
Surfaces of the Rastrigin and Himmelblau functions, respectively, used to evaluate the pso() function of the AdequacyModel package.

The peculiarities of the surfaces of Rastringin and Himmelblau’s functions can be seen in Fig 10(a) and 10(b), respectively. The Fig 10(a) exemplifies situations in which we have an objective function with multiple local minimums and this may cause confusion to many optimization algorithms, specially those based on derivatives. In the second scenario, the pso() function was submitted to a function with four global optimums, in which we have a region of decrease less accentuated, as can be seen in Fig 10(b).

It is important to make it clear that all metaheuristic methods aim to provide the best solution to a problem. However, we cannot guarantee that any optimization method will always find the best solution for complicated functions, such as the Rastringin e Hemmelblau functions. So, we will be satisfied to obtain reasonable solutions for the examples considered here.

Here, we show that the pso() function can be very useful, for example, in the detection of valley patterns on surfaces, since the pso() behavior implemented in the **AdequacyModel** package will make it difficult the attraction of all particles to the same valley, that is, to the same solution candidate to the global minimum. This is probably due to the random substitution of particles leaving the search region, which gives greater variability without removing the precision of the method.

In order to greatly reduce the time of the simulations, the code was implemented to make use of shared memory (multicore) parallelism, using the **parallel** package available in any R installation. In this class of parallelism, we have that the cores are distributed on the same chip. Thus, even if it runs on a computer with more than one processor, only the processor cores that will be running the R environment will be considered. This is sufficient for the simulations in question and will facilitate the checking of the results obtained in this section, since it will significantly reduce the execution time of both simulations when performed on multicore processors that are common today.

Since the iterations of MC are mathematically independent, the idea is to write the loops of MC simulations through the mclaplly() function of the **parallel** package. The mclapply funcional is quite similar to the lapply(), funcional of the base package, where the prefix mc in the functional name refers to the term “multicore”. Unlike lapply(), the functional mcapply() will simultaneously trigger each iteration of the MC loop (thread) over each of the colors of a same processor. In interpreted programming languages, such as the R language, replacing the traditional for repetition structure with a functional one can improve the computational efficiency of the code. The use of functionalities for code repetition is commonplace in programming languages with functional paradigm, which is also one of the paradigms available in the R language.

MC simulations were performed on a computer with an Intel (R) Core i7-4710MQ processor operating between 2.50 GHz (minimum frequency) at 3.50 GHz (maximum frequency), 6 MB cache, 8 threads and 32 RAM memory GB DDR3. For each of the functions, 20 thousand MC simulations were considered. Because it is a randomized methodology, a seed, set.seed(1L), has been set so that the results are reproducible.

To further facilitate the reproduction of the simulations for the considered objective functions, MC simulations can be reproduced by calling the function simulation_mc() which has 8 (eight) arguments:

- mc: number of MC simulations, with standard in mc = 20e3L;
- FUN: can assume the strings “rastrigin” (default) or “himmelblaus” and refers to the objective function considered;
- seed: seed to be considered, where the default is seed = 1L;
- plot.curve: if TRUE (default) will construct the graph with level curves and superimpose the estimated optimal values at each iteration of MC (white dots);
- S: quantity of particles considered, where S = 150 is the standard;
- e: minimum variance of the optimal values of the last iterations, with e = 1e-4 by default;
- N: minimum number of iterations of the PSO method, with N = 50 set by default;
- prop: proportion of the last minimum values that will be considered for the calculated of the variance used as stopping criterion.

With the code attached (see A), the simulations of MC for the Rastrigin (Eq 5) and function of Himmelblau’s (Eq 6) simulations can be reproduced making simulation_mc(mc = 2e4L, FUN = “rastrigin”) and simulation_mc(mc = 2e4L, FUN = “himmelblaus”), respectively. The MC simulation that subjected the pso() function to optimize the Rastringin function took approximately 3.77 hours with the hardwares considered. A much shorter time was required to perform MC simulations for the Himmelblau’s function (Eq 6) because of its simplicity compared to the Rastrigin function (Eq 5), taking about 4.58 minutes.

**Fig 11:**
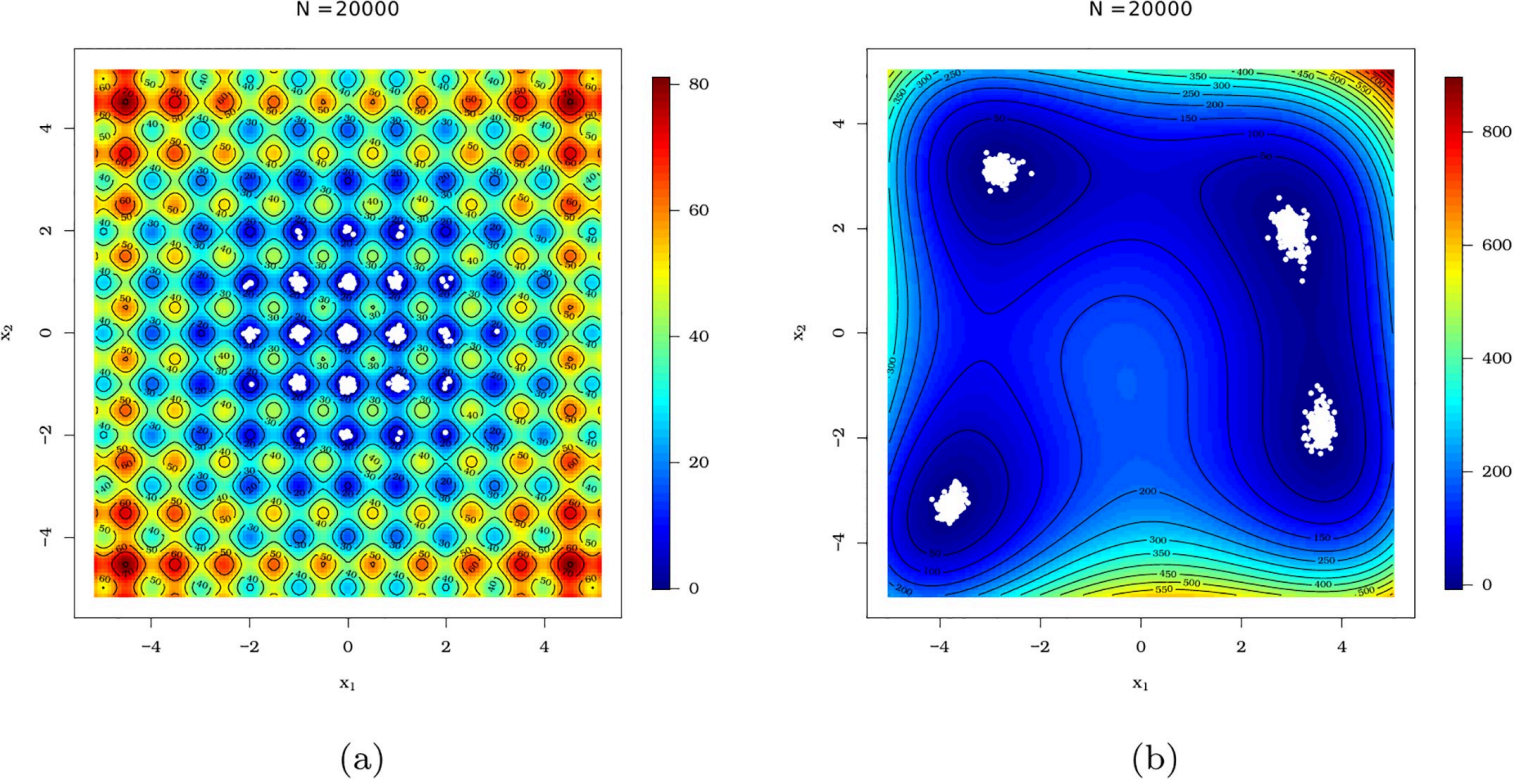
Level curves of the Rastringin and Himmelblau’s functions, respectively, with optimal points (white dots) obtained by the 20 thousand MC simulations (*N* = 20000).

The first MC simulation considered the Rastringin function (Eq 5). The results of each iteration of MC are represented as white dots in the graph of surface level curves, as shown in Fig 11(a). Similarly, each of the 20,000 MC simulations that submitted the pso() function to the optimization process of the Himmelblau’s function (Eq 6) are shown in the graph of the level curves of the function of Himmelblau’s, as can be seen in Fig 11(b).

The PSO algorithm given in Section 2.2 and encoded in the pso() function of the **AdequacyModel** package presented satisfactory results in obtaining global minimums in both cases. For the case of the Rastringin function (Eq 5), not always the best solution is obtained, but good solutions are reached. The dot cloud, as shown in Fig 11(a), has been concentrated in regions with good candidates to the point of global minimum.

The function pso() also provides good results when submitted to the optimization of the Himmelblau’s function (Eq 6). In this case, particles are attracted to the valleys containing the four global optimum points. In addition, it has been realized that the pso() function may be useful in detecting cavities on a surface (valleys detection). This is most likely due to the fact that particles are randomly replaced in the search space, which allows the particles to divide into groups that will not necessarily be attracted to the same cavity. In the situation where there is a mathematical representation of the image of a surface, the pso() function of the **AdequacyModel** package may be useful in detecting cavities (valleys). In addition, the pso() function returns a history of global optimal values that may be useful in detecting these regions.

## 6 Conclusions

In this paper we describe the **AdequacyModel** package developed for the R statistical environment with an easy-to-use set of statistical measures to assess the adequacy of lifetime models for a given data set using the PSO as the underlying optimization method. Our contribution to the PSO is to give more control over some aspects of the algorithm, such as the number of particles and iterations and a stop criterion based on the minimum number of iterations and the variance of a given proportion of optimal values. Simulation studies show that the results obtained by the PSO are not affected by perturbation in initial values. Regarding data analysis, our proposed package allows for easy entering of a data set. Further, the goodness.fit() function provides measures that allow comparison of non-nested models models using the classic AIC, CAIC, BIC statistics. Two empirical applications were presented in order to illustrate the importance and usefulness of the proposed package.

In the future version of the package, the function pso() will be rewritten using the C/C++ language. Rewriting the function in C/C++ will bring benefits regarding the computational performance of the function, since depending on the values of the N, e and prop parameters of the pso() function, we can easily encounter computationally intensive situations.

## A Monte Carlo simulation code

#!/usr/bin/env Rscript

# install.packages(“purrr”)

# install.packages(“AdequacyModel”)

# install.packages(“plot3D”)

# install.packages(“fields”)

# install.packages(“parallel”)

# Monte Carlo Simulation --------------------------------------------------

simulation_mc <- function(mc = 20e3L, FUN = “rastrigin”,

           seed = 1L, plot.curve = TRUE,

           S = 150, e = 1e-4, N = 50L,

           prop = 0.1){

 if (FUN != “rastrigin” && FUN != “himmelblaus”)

 stop(“The argument ”, FUN, ” It is not valid.\n

    Choice \“rastrigin\” or \“himmelblaus\””)

 if (FUN == “rastrigin”){

 # Rastrigin Function ----------------------------------------------------

 obj <- function(par, x, A = 10L) {

  expr_to_eval <-

  purrr::map(.x = 1:length(par),

  .f = ~ parse(text = paste(“x”, .x, “ <- par[”, .x, “]”, sep = “”)))

  x_vector <- NULL

  for (i in 1:length(par)) {

    eval(expr_to_eval[[i]])

    x_vector[i] <- eval(rlang::parse_expr(paste(“x”, i, sep = “”)))

  }

  return(A * length(x_vector) +

  sum(x_vector ^ 2 − A * cos(2 * pi * x_vector)))

 }

 args <- list(

     func = obj,

     S = S,

     lim_inf = rep(-5.12, 2),

     lim_sup = rep(5.12, 2),

     e = e,

     N = N,

     prop = prop

   )

 } else {

 # Hummelblaus Function: -------------------------------------------------

 obj <- function(par, x) {

  x <- par[1]

  y <- par[2]

  (x ^ 2 + y − 11) ^ 2 + (x + y ^ 2 − 7) ^ 2

 }

 args <- list(

     func = obj,

     S = S,

     lim_inf = rep(-5, 2),

     lim_sup = rep(5, 2),

     e = e,

     N = N,

     prop = prop

   )

}

 # One step (Monte Carlo)

 onestep <- function(x, list_args) {

 result <-

  do.call(getExportedValue(“AdequacyModel”, “pso”), args = list_args)

 list(par = result$par, value = result$f[length(result$f)])

 }

 # A combined multiple-recursive generator’ from L’Ecuyer (1999),

 # each element of which is a feedback multiplicative generator with

 # three integer elements: thus the seed is a (signed) integer vector of

 # length 6. The period is around 2^191.

 set.seed(seed = seed, kind = “L’Ecuyer-CMRG”)

 time <- system.time(

  results_mc <-

  parallel::mclapply(

  X = 1:mc,

  FUN = onestep,

  mc.cores = parallel::detectCores(),

  list_args = args

  )

 ) # End system.time().

 results <- unlist(results_mc)

 par_1 <- results[names(results) == “par1”]

 par_2 <- results[names(results) == “par2”]

 value <- results[names(results) == “value”]

 if (plot.curve && FUN == “rastrigin”){

 rastrigin_plot <- function(x,y){

  20 + (x ^ 2 − 10 * cos(2 * pi * x)) +

  (y ^ 2 − 10 * cos(2 * pi * y))

 }

 M <- plot3D::mesh(seq(-5.12, 5.12, length.out = 500), seq(-5.12, 5.12, length.out = 500))

 x <- M$x; y <- M$y

 pdf(file = “monte_carlo_rastrigin.pdf”, width = 9,

   height = 9, paper = “special”,

   family = “Bookman”, pointsize = 14)

   z <- rastrigin_plot(x, y)

   fields::image.plot(x, y, z, xlab = bquote(x[1]), ylab = bquote(x[2]), main = paste0(“N =”, length(par_1)))

   contour(seq(-5.12, 5.12, length.out = nrow(z)), seq(-5.12, 5.12, length.out = nrow(z)), z, add = TRUE)

   points(par_1, par_2, pch = 20, col = rgb(1, 1, 1))

 dev.off()

 }

 else if (plot.curve && FUN == “himmelblaus”){

  himmelblaus_plot <- function(x, y){

   (x ^ 2 + y − 11) ^ 2 + (x + y ^ 2 − 7) ^ 2

  }

 M <- plot3D::mesh(seq(-5, 5, length.out = 500), seq(-5, 5, length.out = 500))

 x <- M$x; y <- M$y

 pdf(file = “monte_carlo_himmelblaus.pdf”, width = 9,

   height = 9, paper = “special”, family = “Bookman”,

   pointsize = 14)

  z <- himmelblaus_plot(x, y)

  fields::image.plot(x, y, z, xlab = bquote(x[1]), ylab = bquote(x[2]), main = paste0(“N =”, length(par_1)))

  contour(seq(-5, 5, length.out = nrow(z)), seq(-5, 5, length.out = nrow(z)), z, add = TRUE, nlevels = 30)

  points(par_1, par_2, pch = 20, col = rgb(1, 1, 1))

 dev.off()

 }

 list(x = par_1, y = par_2, value = value, time = time)

}

# Saving Results ----------------------------------------------------------

result_rastrigin <- simulation_mc(mc = 2e4L, FUN = “rastrigin”)

save(file = “simulation_rastrigin.RData”, result_rastrigin)

result_himmelblaus <- simulation_mc(mc = 2e4L, FUN = “himmelblaus”)

save(file = “simulation_himmelblaus.RData”, result_himmelblaus)
